# Are Sedentary Behaviors Associated with Sleep Duration? A Cross-Sectional Case from Croatia

**DOI:** 10.3390/ijerph16020200

**Published:** 2019-01-12

**Authors:** Lovro Štefan, Maja Horvatin, Mario Baić

**Affiliations:** Faculty of Kinesiology, University of Zagreb, 10 000 Zagreb, Croatia; maja.horvatin@kif.hr (M.H.); mario.baic@kif.hr (M.B.)

**Keywords:** screen-time, short sleep, long sleep, young adults, associations

## Abstract

Although both sedentary behavior and sleep duration are risk factors for obesity, little evidence is provided regarding their mutual associations in young adults, who are at extreme risk of spending more time sitting and having irregular sleeping hygiene. Thus, the main purpose of the present study was to explore the associations between different sedentary behaviors and sleep duration. In this cross-sectional study, we recruited 2100 university students from the city of Zagreb. To assess sedentary behaviors and sleep duration, we used validated questionnaires. The associations between sedentary behaviors and sleep duration were analyzed using logistic regression analyses and were adjusted for sex, body-mass index, self-rated health, socioeconomic status, smoking status, binge drinking, psychological distress and chronic disease/s. Participants being in the third (OR = 1.45; 95% CI 1.05 to 2.01) and fourth (OR = 1.82; 95% CI 1.26 to 2.61) quartile of the screen-time, in the third (OR = 1.49; 95% CI 1.05 to 2.13) and fourth (OR = 1.72; 95% CI 1.22 to 2.42) quartile of the leisure-time sedentary behavior and in the fourth (OR = 1.45; 95% CI 1.04 to 2.02) quartile of the total sedentary behavior were more likely to be ‘short’ sleepers (<7 h). Also, participants being in the third (OR = 1.63; 95% CI 1.16 to 2.30) and fourth (OR = 1.93; 95% CI 1.33 to 2.81) quartile of the screen-time and in the fourth (OR = 1.45; 95% CI 1.05 to 2.00) quartile of the total sedentary behavior were more likely to be ‘long’ sleepers (>9 h). Our study shows that sedentary behavior in screen-time and total sedentary behavior are associated with both ‘short’ and ‘long’ sleep duration.

## 1. Introduction

Sedentary behavior has become a public health problem worldwide. It is defined as a behavior/s where energy expenditure is ≤1.5 metabolic equivalents and is distinguished from time spent sleeping [1]. Independent of physical activity, sedentary behaviors have been associated with negative health outcomes, including overweight/obesity status, elevated blood pressure and total cholesterol, lower levels of self-esteem, physical fitness and academic achievement [2]. Studies have shown that sedentary behavior has many sub-domains, such as watching television, playing computer games, browsing the Internet, reading, and engaging in passive transport [3].

Recently, great attention has been given to exploring the associations between sedentary behaviors and sleep duration [4]. Sleeping <7 h or >8 h is associated with higher rates of mortality [5], as well as with cardiovascular and metabolic diseases [6,7]. Previous findings have reported that inadequate sleep quality (sleep deprivation) is associated with both short- and long-term consequences [8]. Specifically, short term consequences of sleep deprivation are increase stress levels, reduced quality of life, psychological distress and performance deficits, yet long-term consequences are higher incidence of cardiovascular, metabolic and mental diseases and all-cause mortality [8]. Although sedentary behavior consists of many sub-domains, only screen-time exposure (assessed as a proxy of sedentary behavior) has been associated with decreased sleep in many countries among children and adolescents [9]. In this population specifically, the link between sedentary behaviors and sleep duration shows that (1) the time spent in front of the screen can be replaced with sleep [10], (2) the use of media can potentially increase alertness, leading to disturbed sleep [10] and (3) emitted light from the screen may affect sleep by modifying melatonin production [10]. However, previous evidence in adults has shown no association between sedentary behaviors and sleep duration [11,12,13]. Most recently, Lakerveld et al. [14] conducted the study among 6037 European adults which showed similar results to the previous one, where no significant associations between sleep duration and total or other domains of sedentary behaviors were found.

To the best of authors’ knowledge, no study has yet explored the associations between different domains of sedentary behaviors and sleep duration in young adults. A recent study showed that young adults spent approximately 64 h/week (9.1 h/day) in sedentary behaviors [15], yet 18.1% and 17.1% of them were ‘short’ and ‘long’ sleepers [16]. Also, in a majority of countries, adolescents/young adults ≥19 years of age start university, move out of their parents’ home or start work, which leads to higher amount of psychological distress, extensive electronic media use and disturbed sleep [17]. Therefore, the population of young adults is at extreme risk engaging in different domains of sedentary behavior and having irregular sleep hygiene.

Thus, the main purpose of the present study was to explore the associations between different domains of sedentary behaviors (screen-time, at the faculty, leisure-time and total) and sleep duration.

## 2. Materials and Methods

### 2.1. Study Participants

This cross-sectional study was based on a sample of university students from the city of Zagreb. Detailed procedure and sample collection are reported elsewhere [16,18,19]. In brief, we randomly selected eight out of thirty-three faculties from the University of Zagreb in 2017 at the first stage. At the second stage, we contacted a teacher at each faculty to help us with conducting the study. Finally, out of 2320 students that were approached, 2100 of them (90.5%) provided full data and were included in further analyses. All the analyses and procedures were anonymous and in accordance with the Declaration of Helsinki. The Institutional Review Board of the Faculty of Kinesiology (Ethics Code No: 16/2017) approved the study. Also, all participants gave their written informed consent for participation in the study and have agreed to have their data used for scientific purposes.

### 2.2. Outcome Variable

To assess sleep duration, we asked the following questions: ‘What time do you usually go to bed during weekday and weekend day’. The same two questions were asked for ‘get-up time’ during weekday and weekend day [20]. Sleep duration was calculated by subtracting ‘go to bed’ from ‘get up’ time.

Average weekly value of sleep duration was calculated using following equation:

Average SD = [(week day SD × 5) + (weekend day SD × 2)]/7(1)

For the purpose of this study, we divided sleep duration into three categories as follows: (1) <7 h (‘short’ sleepers), (2) 7–9 h (‘normal’ sleepers) and (3) >9 h (‘long’ sleepers). Self-reported sleep duration was previously validated and moderately correlated with actigraphy [21] and is often used to assess sleep duration in young adults [20].

### 2.3. Sedentary Behaviors

To assess sedentary behaviors, we asked participants about the time they spent in six different groups of behaviors in hours and minutes per day: (1) watching television, (2) playing computer games, (3) browsing the Internet, (4) homework and studying, (5) listening to music and (6) reading. We divided the categories into three major domains: (1) screen-time (watching television, playing computer games and browsing the Internet), (2) work/faculty sedentary behaviors (homework and studying) and (3) leisure-time sedentary behaviors (listening to music and reading). Finally, we summed all three domains to obtain the total time spent in sedentary behaviors. Additionally, we included sex, body-mass index, self-rated health, socioeconomic status, smoking status, binge drinking, psychological distress and chronic disease/s as covariates by questionnaire using previously described measures [16,18,19].

### 2.4. Data Analysis

Basic descriptive statistics of the participants are presented as frequencies (N) and percentages (%). First, hours and minutes of sedentary behaviors were converted to minutes. Next, a continuous variable for each domain (screen-time, work and leisure-time sedentary behaviors) was used to categorize the participants into quartiles. Differences between ‘short’, ‘normal’ and ‘long’ sleep in terms of screen-time, leisure-time sedentary behavior, work and total sedentary behavior were calculated using chi-square test. Next, the association between each domain with sleep duration was calculated using generalized estimating equations with logistic regression analyses for faculty clustering. Two sets of logistic regressions were performed in order to calculate odds ratios (ORs) with 95% confident intervals (95% CIs) for both ‘short’ (<7 h) and ‘long’ (>9 h) sleep duration, as compared with a normal sleep duration (7–9 h). Each sedentary behavior domain was separately entered into the model to examine the association with ‘short’ and ‘long’ sleep duration. Additionally, each of eight models (2 sets of regression analyses × 4 domains of sedentary behaviors) was adjusted for sex, body-mass index, self-rated health, socio-economic status, smoking status, binge drinking, psychological distress and chronic disease/s. The interaction term between gender and sedentary behaviors was not statistically significant and we dropped the gender-stratified analyses. Significance was set up at α = 0.05. All the analyses were performed in Statistical Package for Social Sciences Software, V.22 (IBM, Armonk, NY, USA).

### 2.5. Ethical Approval

This study was approved by the Institutional Review Board of the Faculty of Kinesiology, University of Zagreb, Croatia under the number 16/2017.

## 3. Results

Basic descriptive statistics of the study participants are presented in Table 1. In brief, mostly the highest percentage of the participants was in the fourth quartile (the highest) in screen-time, work and total sedentary behaviors. Participants that were categorized in the fourth quartile were in general both ‘short’ and ‘long’ sleepers, compared to ‘normal’ sleepers (*p* < 0.05), with the exception of work sedentary behavior.

**Figure 1:**
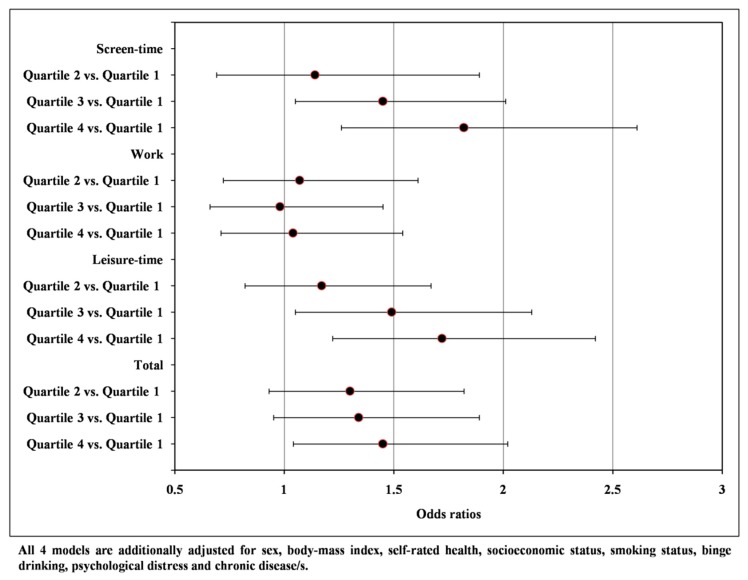
Associations between sedentary behavior domains and ‘short’ sleep duration (<7 h) vs. ‘normal’ sleep duration (7–9 h).

**Figure 2:**
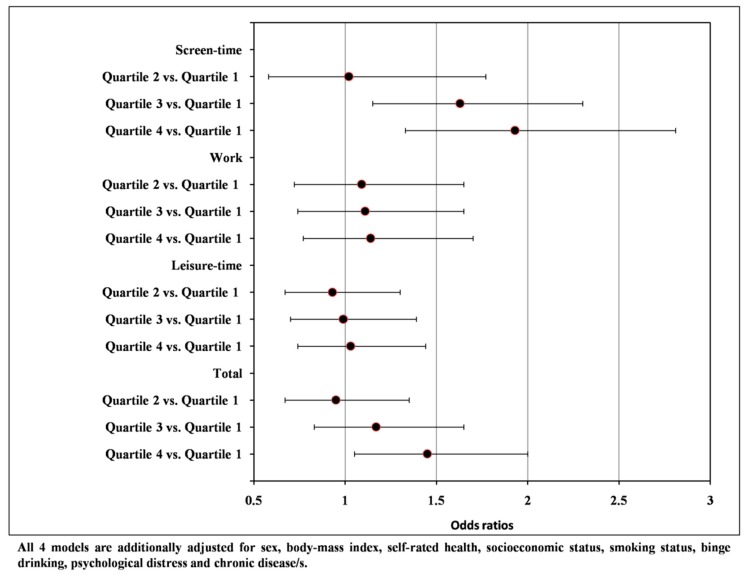
Associations between sedentary behavior domains and ‘long’ sleep duration (>9 h) vs. ‘normal’ sleep duration (7–9 h).

The associations between each of the sedentary behavior domains and ‘short’ and ‘long’ sleep are presented in Figure 1 and Figure 2. Participants categorized in the third (OR = 1.45; 95% CI 1.05 to 2.01) and fourth (OR = 1.82; 95% CI 1.26 to 2.61) quartile of the screen-time, in the third (OR = 1.49; 95% CI 1.05 to 2.13) and fourth (OR = 1.72; 95% CI 1.22 to 2.42) quartile of the leisure-time and in the fourth (OR = 1.45; 95% CI 1.04 to 2.02) quartile of the total sedentary behavior were more likely to be ‘short’ sleepers (<7 h). In the second set of logistic regression analysis, participants categorized in the third (OR = 1.63; 95% CI 1.16 to 2.30) and fourth (OR = 1.93; 95% CI 1.33 to 2.81) quartile of the screen-time and in the fourth (OR = 1.45; 95% CI 1.05 to 2.00) quartile of the total sedentary behavior were more likely to be ‘long’ sleepers (>9 h).

## 4. Discussion

The main purpose of the present study was to examine whether sedentary behavior domains and total sedentary behavior were associated with sleep duration. Our study shows that more time spent in front of the screen, in leisure-time sedentary behavior and in total sedentary behavior is associated with higher likelihood of being a ‘short’ sleeper, yet more time spent in front of the screen and in total sedentary behavior is associated with higher likelihood of being a ‘long’ sleeper.

Our results which show the associations between screen-time and ‘short’ sleep duration are in line with previous studies [10,14,22]. Lakerveld et al. [14] showed that shorter sleep was significantly associated with increased screen-time in a large sample of European adults (*N* = 6037), irrespective of urban region, gender, age, educational level and weight status. As mentioned in the ‘Introduction’ section, the link between screen-time and ‘short’ sleep duration is described by three potential mechanisms as follows: (1) the time spent in front of the screen can be replaced by sleep [10], (2) the use of media can potentially increase alertness, leading to disturbed sleep [10] and (3) emitted light from the screen may affect sleep by modifying melatonin production [10]. Replacing screen-time with sleep may be an effective way to reduce the risk of all-cause mortality [23]. In adolescents, studies have shown similar associations where screen-time is a significant predictor of shorter sleep and daytime sleepiness [24]. From the physiological perspective, the hypothalamic pituitary has a role of maintaining both alertness and sleep functioning. Thus, by engaging in screen-time (especially playing computer games), the level of alertness increases and modifies sleep and circadian rhythm [25].

Our study also shows that screen-time sedentary behaviors are associated with ‘long’ sleep duration, which is not in line with previous studies [10,14,22]. However, although non-significant, Lakerveld et al. [14] showed positive association between screen-time and ‘long’ sleep duration. The mechanism underlying such association is based on the interconnection between screen-time and sleep disturbances. One previous study showed that more time spent in front of the screen is associated with higher levels of sleep disturbances [26]. Moreover, Patel et al. [27] showed that participants reported having restless legs syndrome and those who were snorers were more likely to be categorized as ‘long’ sleepers. Therefore, sleep disturbances may be a potential mediator between screen-time and ‘long’ sleep duration according to our study. However, we did not adjust for sleep disturbances and can only speculate that they might have a significant mediation role between sedentary behavior and sleep duration.

Next, leisure-time sedentary behavior (reading, listening to music) was significantly associated with ‘short’, but not ‘long’ sleep duration. To the best of our knowledge, this was the first study examining the association between leisure-time sedentary behavior and sleep duration in a large sample of university students. We speculate that academic performance, which includes doing homework and studying, was being performed by students—during the evening and before bedtime. To justify, our study was conducted between October and November of 2017 and at this specific period first examinations (first tests) take place at Croatian universities. Also, classes are often scheduled between 8 am and 4 pm and students attend to their academic obligations in their leisure-time. Thus, it is possible that during that period students were more engaged in pursuing their academic obligations, along with their hobbies (playing an instrument) late at night, which could have potentially led to ‘short’ sleep duration.

Finally, our results show that being in the highest quartile of total sedentary behavior is associated with both ‘short’ and ‘long’ sleep duration, after adjusting for numerous covariates. Previous studies have shown no association between total sedentary behaviors and sleep duration [14,28]. Specifically, Lakerveld et al. [14] showed that, although non-significant, total sedentary behavior was positively associated with ‘short’ sleep duration, yet negatively associated with ‘long’ sleep duration. Similar findings were observed in the study by Loprinzi et al. [28]. However, the same study showed that for every 60 min increase in sedentary behavior, participants were 16.0% more likely to almost feel not rested during the day and were 22.0% more likely to feel overly sleepy. The existing association in our study could be explained by the fact that the study was conducted among a relatively homogenous sample of university students from one city, whereas previous studies had been conducted among adult populations. Also, our classification of ‘short’ (<7 h), ‘normal’ (7–9 h) and ‘long’ (>9 h) sleep duration was not the same as in the aforementioned studies. Finally, a relatively high proportion of our participants reported being ‘short’ sleepers (18.1%) and ‘long’ sleepers (17.1%), which might have led to more stable confident interval parameters.

Our study has a few strong points. Firstly, we conducted a study among a representative and large sample of university students (*N* = 2100). Secondly, we adjusted for numerous socio-demographic and health-related covariates.

However, our study also has several limitations. Firstly, a cross-sectional design of the study does not let us conclude the causality of the associations, that is, if ‘short’ and ‘long’ sleep duration lead to more time spent in sedentary behavior. Secondly, we used subjective measures to assess sedentary behaviors and sleep duration, which might have caused possible measurement error and bias. Thirdly, we conducted the study among university students, who do not represent young adults in general and included young adults who do not attend faculties and are currently employed, which could also have led to different results. Finally, the study did not take into account measure of physical activity that could interact with the effects on sleeping (a sporty young person could sleep differently from a non-sporty one with the same hour spent in front a screen).

## 5. Conclusions

In conclusion, our study shows that screen-time, leisure-time and total sedentary behaviors are associated with ‘short’ sleep duration, yet screen-time and total sedentary behaviors are associated with ‘long’ sleep duration in a large sample of university students. This study presents some new evidence about the association between sedentary behaviors and sleep duration. Our study provides some important findings about the aforementioned associations, since both sedentary behavior and sleep duration might be risk factors for obesity and targeting such behaviors should be the purpose for the upcoming strategies and policies reducing the prevalence of obesity through intervention programs. However, future studies should use objective methods (actigraphy) to assess both sedentary behaviors and sleep duration and the nature of the study should be longitudinal, in order to establish causal association.

## Figures and Tables

**Table 1:** Basic descriptive statistics of the study participants (*N* = 2100).

Sedentary Behaviors	Total Sample (*N* = 2100)	‘Short’ Sleepers (*N* = 380)	‘Normal’ Sleepers (*N* = 1360)	‘Long’ Sleepers (*N* = 360)	*p*-Value *
	*n* (%)	*n* (%)	*n* (%)	*n* (%)	
Screen-time					<0.001
Quartile 1	400 (19.0)	61 (16.1)	288 (21.2)	51 (14.2)	
Quartile 2	166 (7.9)	28 (7.4)	117 (8.6)	21 (5.8)	
Quartile 3	973 (46.3)	180 (47.4)	619 (45.5)	174 (48.3)	
Quartile 4	561 (26.7)	111 (29.2)	336 (24.7)	114 (31.7)	
Work					0.532
Quartile 1	259 (12.3)	45 (11.8)	172 (12.6)	42 (11.7)	
Quartile 2	506 (24.1)	92 (24.2)	327 (24.0)	87 (24.2)	
Quartile 3	629 (30.0)	109 (28.7)	412 (30.3)	108 (30.0)	
Quartile 4	706 (33.6)	134 (35.3)	449 (33.0)	123 (34.2)	
Leisure-time					0.031
Quartile 1	462 (22.0)	64 (16.8)	314 (23.1)	84 (23.3)	
Quartile 2	569 (27.1)	93 (24.5)	381 (28.0)	95 (26.4)	
Quartile 3	506 (24.1)	99 (26.1)	322 (23.7)	85 (23.6)	
Quartile 4	563 (26.8)	124 (32.6)	343 (25.2)	96 (26.7)	
Total					0.037
Quartile 1	498 (23.7)	75 (19.7)	344 (25.3)	79 (21.9)	
Quartile 2	523 (24.9)	99 (26.1)	348 (25.6)	76 (21.1)	
Quartile 3	487 (23.2)	91 (23.9)	312 (22.9)	84 (23.3)	
Quartile 4	592 (28.2)	115 (30.3)	356 (26.2)	121 (33.6)	

* Bivariate chi-square test.
